# Supplementary material for: Interplay between social influence and competitive strategical games in multiplex networks
Source: Sci Rep. 2017 Aug 1;7:7087. doi: 10.1038/s41598-017-06933-2 (PMC5539333; doi:10.1038/s41598-017-06933-2)
Supplement: Supplementary file 1 — Supplementary Materials [file 41598_2017_6933_MOESM1_ESM.pdf]

Supplementary Materials for ”Interplay between social influence and competitive strategical games in multiplex networks”

Roberta Amato,[1,2] Albert Díaz-Guilera,[1,2] and Kaj-Kolja Kleineberg[3,*]

[1]Department of Physics, University of Barcelona, Martí i Franquès 1, E-08028, Barcelona, Spain
[2]Institute of Complex Systems (UBICS), Universitat de Barcelona, Martí i Franquès 1, E-08028, Barcelona, Spain
[3]Computational Social Science, ETH Zurich, Clausiusstrasse 50, CH-8092 Zurich, Switzerland

I. FINAL COOPERATION

In Fig. 1 we show the final density of cooperators averaged over 50 realization of the system.

II. PHASE DIAGRAMS FOR NUMERICAL SIMULATIONS

Fig. 2 shows the probability to reach the harmony state from different initial conditions. The region in which the final state differs for the different initial conditions is the bistable region.

III. MEAN FIELD EQUATION DERIVATION

Here, we show how one can derive the mean field equations for the coupled dynamics.

For the N nodes of the system, let us assign a state σ_i^α to each node i $(i = 1, 2, \dots, N)$ in the layer α $(\alpha = I, II)$ such that $\sigma_i^\alpha = 1(0)$ if the node has the strategy, respectively opinion, $C(D)$ in the given layer α. In the GN, the probability for a node i to copy the strategy of one of her random selected neighbor j is given by

$$P_{i \leftarrow j} = \frac{1}{2} \left(1 - \tanh\left[\pi_i - \pi_j\right]\right) . \tag{1}$$

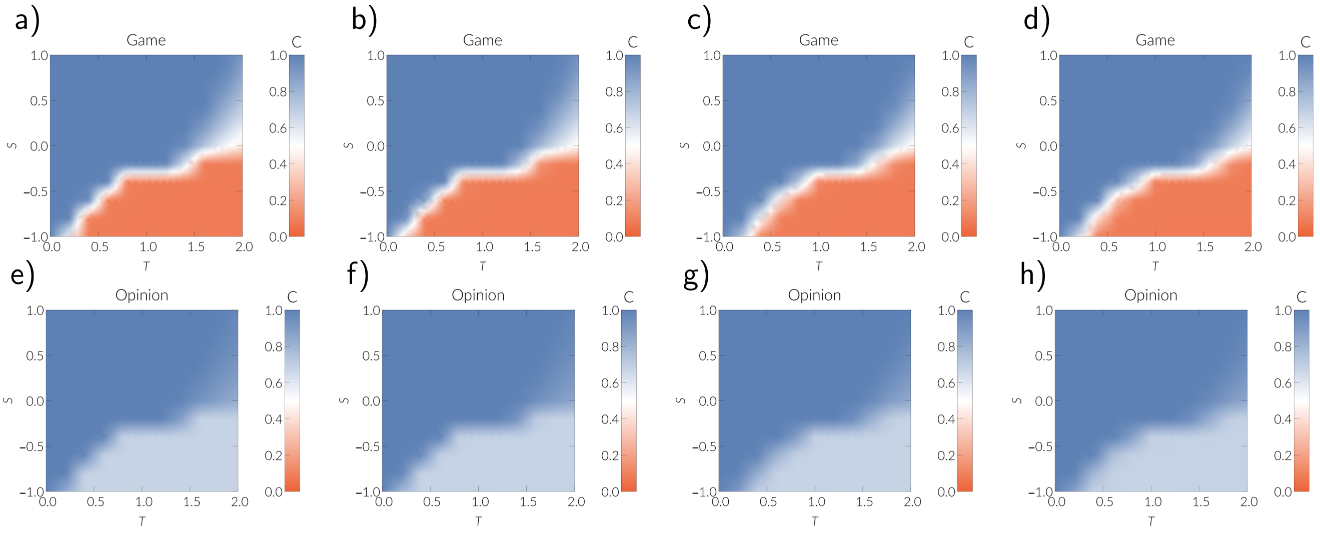

Figure 1: Final density (after 5×10^5 rounds) of cooperation for different correlation values. **(a, e)** Uncorrelated ($g = \nu = 0$). **(b, f)** Radial correlations ($g = 0, \nu = 1$). **(c, g)** Angular correlations ($g = 1, \nu = 0$). **(d, i)** Angular and radial correlations ($g = 1, \nu = 0$). Game layer is shown in the top row **(a-d)**. Opinion layer is shown in the bottom row **(e-h)**.

*Electronic address: `kkleineberg@ethz.ch`

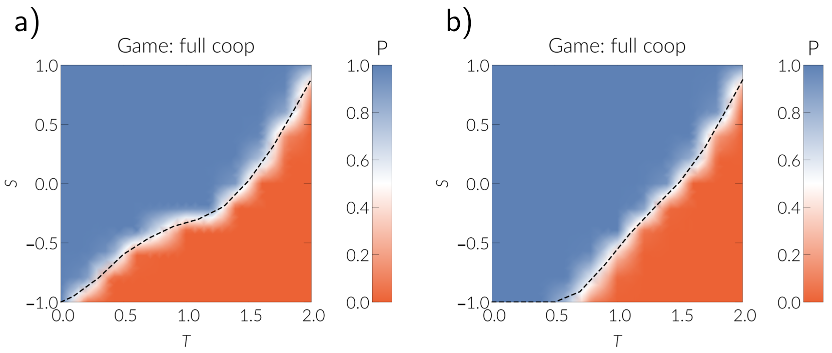

Figure 2: Results for GMM multiplexes with $\gamma = 0.2$, $\beta = 0.7$, $N = 10000$ nodes, $g = 1$, and $\nu = 0$. **(a)** Probability to reach the harmony state starting with $C_{I,II} = 0.01$ and $g = 1, \nu = 0$. **(b)** The same for starting with $C_{I,II} = 0.99$.

Each player's payoff can be calculated from the payoff matrix

$$M = \begin{array}{c|cc} & \text{C} & \text{D} \\ \hline \text{C} & 1 & S \\ \text{D} & T & 0 \end{array} . \tag{2}$$

In particular, each player plays $\langle k \rangle$ games with randomly chosen opponents, where $\langle k \rangle$ is the mean degree in layer I. The average payoff for cooperators, c_I, and defectors, d_I, is then given by

$$\begin{aligned} \pi_{c_I} &= \langle k \rangle c_I + \langle k \rangle S (1 - c_I) \\ \pi_{d_I} &= \langle k \rangle T c_I . \end{aligned} \tag{3}$$

The transition probabilities corresponding to the game dynamics are expressed by

$$\begin{aligned} w_I(D \to C) &= \frac{1}{2} c_I \left(1 - \tanh\left[\pi_{d_I} - \pi_{c_I}\right]\right) \\ w_I(C \to D) &= \frac{1}{2} (1 - c_I) \left(1 - \tanh\left[\pi_{c_I} - \pi_{d_I}\right]\right) , \end{aligned} \tag{4}$$

for which the term regarding the game dynamics in the evolution of cooperators of layer I is

$$\begin{aligned} \partial_t c_I &= (1-\gamma)\left\{(1 - c_I)\, w(D \to C) - c_I w(C \to D)\right\} + \gamma \mathcal{C}_{I,II} \\ &= (1-\gamma) c_I \left(1 - c_I\right) \tanh\left[\langle k \rangle \left(c_I(1-T) + S(1-c_I)\right)\right] \\ &+ \gamma \mathcal{C}_{I,II} , \end{aligned} \tag{5}$$

where $\mathcal{C}_{I,II}$ denotes the coupling term between the game and opinion dynamics. The dynamics in the opinion layer, II, is described as a biased voter model for the diffusion of opinions with a bias $\beta \in (0,1)$ towards the "cooperate" opinion. A node i will then adopt the state of one of its randomly selected neighbors j with probability β if $\sigma_j^{II} = 1$ and $1 - \beta$ if $\sigma_j^{II} = 0$. The voter model dynamic is given by

$$\partial_t c_{II} = (1-\gamma)(2\beta - 1) c_{II}(1 - c_{II}) + \gamma \mathcal{C}_{II,I} . \tag{6}$$

The interaction term is given by

$$\mathcal{C}_{I,II} = (c_{II} - c_I) \tag{7}$$

$$\mathcal{C}_{II,I} = (c_I - c_{II}) , \tag{8}$$

meaning that a node copies her status from the respective other layer. Combining the equations yields

$$\begin{aligned} \partial_t c_I =& (1-\gamma) c_I (1 - c_I) \tanh\left[\langle k \rangle \left(c_I(1-T) + S(1-c_I)\right)\right] + \gamma(c_{II} - c_I) \\ \partial_t c_{II} =& (1-\gamma)(2\beta - 1) c_{II}(1 - c_{II}) + \gamma(c_I - c_{II}) . \end{aligned} \tag{9}$$

IV. IMPACT OF THE COUPLING CONSTANT γ

In Fig. 3 we show the bifurcation diagram with the coupling strength γ as a control parameter. At a critical value $\gamma_c \approx 0.4$ the system undergoes a transcritical bifurcation. For $0 < \gamma < \gamma_c$ we have a stable mixed solution, which is particularly interesting. In the main text, we therefore fix γ in this range.

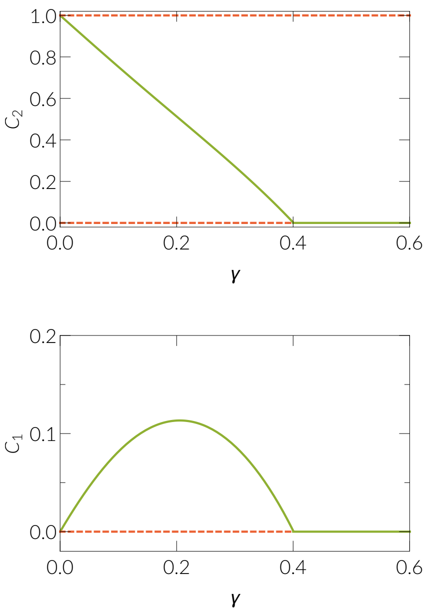

Figure 3: Bifurcation diagram for the prisoner's dilemma ($T = 1.5, S = -0.5$) for $\beta = 0.7$ as a function of the control parameter β. Top: Density of cooperative attitude in the opinion layer. Bottom: Density of cooperators in the game layer.
